# Patient interviewing strategy and tactics in teaching professional foreign languages for medical purposes

**DOI:** 10.25122/jml-2021-0252

**Published:** 2022-04

**Authors:** Yulia Biryukova, Victoria Kurilenko, Yulia Gosteva, Kristina Klasnja, Marina Bragina

**Affiliations:** 1. Russian Language Institute, Peoples' Friendship University of Russia (RUDN University), Moscow, Russia

**Keywords:** teaching foreign language for medical purpose, methodological strategy, stages of patient interviewing, doctor's speech skills

## Abstract

Communication skills are critical abilities that medical students need in their practice and further work, especially in interviewing patients. Interviews conducted efficiently are the basis for prescribing appropriate treatment and recovery. In this article, we presented methods and strategies of teaching foreign languages for developing skills and abilities in interviewing patients (professional purposes). The research was conducted from 2016 until 2018 in two groups of medical students studying Russian as a foreign language. In the first group, the teaching was carried out according to the strategy proposed by the authors. In the second group, the foreign language was taught according to the curriculum. The total number of students was 96 in both groups. The average of the total score and studying dynamics were calculated. Students in the first group with a three-stage methodological algorithm were more successful in finishing the course than the second group. The results section shows that the standard model of teaching the Russian language for international medical students can lead to misunderstanding and misreporting the current diagnosis. However, the strategy described in this article can be considered an effective algorithm for teaching international medical students.

## Introduction

Teaching medical students a foreign language is important because of intensive cooperation between medical specialists and representatives of various countries and cultures. Colleagues actively discuss various clinical cases and draw conclusions about the causes and conditions of diseases, thereby promoting and developing the world of science. Moreover, the analysis of clinical cases shows that bilingual doctors who can communicate directly with patients and their relatives achieve better results in diagnosing and treating diseases [1].

Direct communication is reflected in interviewing patients, which plays a key role in diagnosis, prescriptions of appropriate treatment, and positive prognosis for recovery. Therefore, it is necessary to develop the professional foreign language competence of future doctors in practice to use this knowledge and skills in their professional activities. In this regard, the question arises of how to effectively build the training of medical students in foreign-language interviewing and which training strategy to choose. 

The problems of teaching a foreign language for professional purposes are highlighted in the works of several researchers [2–7]. These authors emphasize that learning a foreign language should be considered a means of receiving additional professional knowledge and developing important qualities. To develop a training course, the authors should consider that it should be systematic and justifiable and begin with understanding its goals and objectives [8–10]. Materials are supposed to be selected and developed thoroughly. Texts should be authentic and updated to develop students' skills. Students should learn terms and grammar to comprehend these texts. Moreover, to develop professional competence, special technologies are required [2–4, 11–14]. Teaching medical language in a foreign language is the subject of research conducted by various methodologists. They all suggest that the training system should have a clear purpose, assessment, material selection and development, teaching, and program evaluation [2].

The analysis of various educational materials devoted to learning a foreign language for medical purposes shows that there are many courses that local communities can use in hospitals, clinics, and doctor's offices. Wei La proposes a Mandarin Chinese course for nursing students to address the needs of the local Mandarin-speaking community. The course is developed for elementary learners of Mandarin. During this course, nursing students learn to construct a list of sentence patterns, words, and phrases frequently used in the medical environment. Authentic materials (forms, documents, signs, posters etc) are used in the course. For speech role-playing games, scenarios are prepared with consultations from nursing professors, in-service nurses, doctors, and patient volunteers. At the end of the course, students pass a final examination in the form of controlled role-playing case scenarios, translations, and a written test on cultural knowledge. Students are also asked to fill out a form to leave their feedback on the course and conduct a self-evaluation of their results [2, 5]. 

Sara Hillman presents a course for in-service healthcare professionals with no previous knowledge of Arabic [15]. The course introduces the basic language for nurses, doctors, and cultural knowledge of Arabic medical traditions. One of the main problems of this course is that the number of contact hours is very limited. In addition, the very nature of Arabic makes this language difficult for native English speakers to learn. Therefore, the author suggests stressing basic spoken Arabic language skills to help put patients at ease. The field of interest is the study conducted by Anna Szawara for a course in Polish for Health Personnel meeting the needs of local hospitals and clinics in Illinois, where there are many Polish-speaking patients. The author suggests the following learning outcomes. Students should be able to correctly pronounce the letters of the Polish alphabet and write down Polish names or words following the speakers:

• Name parts of the body to patients in the target language;
• Instruct patients using basic commands;
• Find out patient conditions;
• Identify patient problems and report them to the doctor;
• Schedule follow-up patient visits [10].

There are many medical English courses, and much attention is paid to studying specialized medical terminology and collocations, reading medical texts, and teaching doctor-patient communication from history assessment and examination to diagnosis and treatment [16–23].

Thus, the analysis of existing medical foreign-language courses based on different languages shows that a large amount of time is given to teaching how to interview patients. Medical professionals working with patients who speak different languages should be able to interview their patients to determine their diseases, provide treatment, and explain how to maintain health.

### Doctor's professiogram: interviewing patients

To keep a comprehensive medical history, the doctor should be calm, attentive, empathetic, and polite. The patient, especially a foreigner, who has a different cultural background, may behave differently than the cultural attitudes of the doctor. These differences cover the paralinguistic aspects of communication, including volume or loudness, tone, pitch, speech rhythms, delivery rate, pausing before responding, sentence stress, intonation, and flow. Moreover, the doctor should pay attention to the patient's facial expression, eyes, and hand gestures. All this can give a clue to what remains unspoken and make a correct diagnosis. It should be added that the doctor must express empathy. The patient must feel that the medical specialist understands his/her case and treats it as unique. All these factors provide a framework for systematically analyzing several components of communicative acts: the source of the communication, the message, and the audience that require attention during the studying and practicing [24]. The doctor must prepare questions in advance. They may be open- or close-ended questions, leading questions, or detailed questions. The type of questions to choose depends on the doctor's information. The questions should be changed one by one by default [25, 26]. This point is rather significant. The patient needs time to give a more thoughtful and accurate response. Sometimes doctors do not keep these rules in mind, and confidence in doctor-patient communication is blocked. Conversations with practitioners show that the reasons may be as follows. Among the main reasons is the doctor's authoritarianism in response to the patient's self-disclosure. In addition, the process of interviewing can be complicated by the following factors:

1. The doctor involuntarily suggests the symptoms of an assumed disease to the patient. V.A. Uryvaev explains the mechanism of such suggestion as follows: the doctor develops a certain hypothesis regarding the nature of the disease and unconsciously tries to fit the actual data to this hypothesis, with the result that suggestive tactics of discourse are misused:
  Doctor: Do you have a headache?
  Patient: No, I don't.
  D.: Have you ever had headaches?
  P.: Well, I cannot say so categorically.
  D.: So, do you have headaches or not, after all?
  P.: Well, I think I do.
  D.: Please, be more precise [27].
2. P.: As a matter of fact, I do; mismatch of communication codes: the doctor, unskilled in the inter-style transformation of linguistic means, begins to ask the patient who does not have a special education such questions as: Where does the pain irradiate? What is an attack of pain stopped by? etc;
3. Inability to recognize aggravation and, especially, simulation of symptoms. To prevent these phenomena, the doctor should be well aware of patient interviewing strategies, be able to ask for clarifications, suggestions, and other questions;
4. Inattention to the patient, lack of empathic listening skills, unwillingness, and inability to listen to the patient during interviewing or consulting.

Patient interviewing has specific features that a specialist should know. First, the doctor should be competent in communication with the patient. To obtain all the necessary information from the patient, the doctor is supposed to know the whole structure of the interview from the beginning to the end.

### Patient interviewing strategy and tactics: doctor's speech skills

The patient interviewing strategy is implemented through a set of tactics that will allow the doctor to have an idea of the patient's condition and make a correct diagnosis. Conversations, scientific research of doctors allowed us to single out the following tactics: information request, information delivery, interlocutor impact, and active empathic listening [28]. In the proposed paper, the authors considered detailed tactics and highlighted the skills that the doctor must possess to carry out successful communication.

1. Information request tactics, *i.e.*, he should have the ability to ask questions of all necessary types; to give adequate speech reactions; to use universal tactics for harmonizing discursive interaction etc, in accordance with the situation and the interlocutor's characteristics;
2. Information delivery tactics, *i.e.*, the primary means of their implementation is a "monologue in dialogue": brief consultations in interviewing the patient, which requires the following skills;
3. Discursive interlocutor impact tactics mean that the doctor should be able, following the situation and the interlocutor's characteristics, to combine the persuasion, information delivery and/or explanation tactics; motivation, information delivery and/or explanation tactics etc;
4. Active empathic hearing tactics (one of the most important components of interviewing, which has not yet received sufficient coverage in professional-oriented linguistic didactics). Active empathic listening is a complex discursive activity, which involves tactics of active detailed listening to monologic speech; tactics of critical interactive listening to dialogical speech; and basic skills in combining the tactics of active detailed and critical interactive listening.

Thus, the range of tactics and speech skills that a doctor should possess is quite broad. However, to successfully implement these tactics, it is obvious that a future foreign specialist must have the skills mentioned above. Therefore, when teaching a professional foreign language to medical specialists, the teacher should pay special attention to developing these skills so that future doctors can easily communicate with their patients. 

The analysis of textbooks shows that a typical scheme of teaching the patient interviewing strategy and tactics is reduced to memorizing dialogues by heart or performing separate communication exercises. As a rule, the patient interviewing strategy is not fully presented to students.

It is necessary to organize the training of international students and future doctors around the patient interviewing strategies in several stages.

1. Introducing a typical staged deployment scheme of the interviewing strategy.
2. Acquiring necessary linguistic means of implementing the interviewing strategy.
3. Involving a role-playing game to imitate real doctor-patient communication.

Let us take a closer look at each of these stages and give specific examples taken from textbooks for medical students studying medical Russian. 

The interviewing strategy may vary depending on the symptoms and the disease itself. For example, if the patient complains of a cough, this can indicate some problems with the respiratory system. Moreover, if the patient has pain in the epigastric region, then the final diagnosis will be associated with problems in the gastrointestinal tract. Each disease of different body systems will have its symptoms and, therefore, interviewing will have its specifics. For comparison, it is possible to consider the schemes of the strategy for interviewing patients with problems of the respiratory system and problems of the gastrointestinal tract (Table 1).

**Table 1.** The difference in strategies for interviewing patients with different diseases [28].

**The strategy for interviewing patients with diseases of the digestive system**	**The strategy for interviewing patients with diseases of the respiratory system**
1. Chief complaints 2. Character of cough 2.1. With sputum/without sputum 2.2. Intensity 2.3. Duration 2.4. Time of occurrence 3. Character of sputum 3.1. Colour 3.2. Consistency 3.3. Amount 3.4. Time of occurrence 4. Pains 4.1. Presence of pains 4.2. Pain localisation 4.3. Conditions for pain occurrences 5. Other symptoms 5.1. Laboured breathing 5.2. Sweating 5.3. Sleep and appetite	1. Chief complaints 2. Characteristics of pain 2.1. Localisation 2.2. Character 2.3. Duration 2.4. Repetition 3. Conditions for pain occurrences 3.1. Relationship with meals 3.2. Relationship with food products 3.3. Time of pain occurrence 4. Dietary pattern 5. Other complaints 5.1. Nausea 5.2. Vomiting 5.3. Heartburn 5.4. Eructation 5.5. Appetite 5.6. Stool

Then, students were presented with a comprehensive doctor-patient dialogue with all possible questions and answers. Let us see a fragment of a comprehensive dialogue-interview with a patient with symptoms of respiratory disease (Tables 2–5).

**Table 2.** A fragment of a dialogue with a patient with symptoms of respiratory disease [28].

**Doctor's questions**	**Patient's possible answers**
**1. Chief complaints** – What seems to be your trouble?	– I have a fever, my ears are ringing. And I feel shivery. I probably caught a cold yesterday, I was in a fever all night, and I had a bit of a rasped throat. And today it hurts me to swallow, my nose is stuffed up, and a cough has begun
**2. Character of cough**	
**a) with sputum/without sputum:**	
– Which cough do you have: dry or wet?	– The cough is wet (dry) – The sputum is not coughed up
**b) intensity:**	
– Which cough do you have: slight or heavy?	– Not so heavy/I cough very heavily
**c) frequency:**	
– Is your cough persistent or attack-like?	– I have attacks of coughing – I cough almost constantly
**d) time of occurrence:**	
– When does your cough occur? – When do you cough: by day or at night? – When do you cough more: in the morning or in the evening?	– As a rule, in the morning, and in the evening it increases – More in the evening\morning – I do not cough at night

**Table 3.** An example of exercises aimed at word building [28].

**Exercise 1. Продолжите ряд/Complete the list of words:**
болезненный (sickly) – болезненность (sickliness), охриплый (hoarse) –…, потливый (sweaty) – …, сонливый (sleepy) – …; нарушить (violate) – нарушение (violation), мучить (trouble) – …, потеть (sweat) – …, першить (have a scratchy throat) – …, напрягаться (г/ж) (strain oneself) – …, затруднять (complicate) – …, потемнеть (tarnish) – ...
**Exercise 2. Подберите однокоренные глаголы к следующим существительным/Match single-root verbs to the following nouns:**	
вдох (inhale) – …, выдох (exhale) – …, ходьба (walk) – …, кашель (cough) – …, подъём (по лестнице) (climb (up the stairs)) – ….

**Table 4.** An example of finding synonyms/antonyms [28].

**Exercise 1. Найдите антонимы к прилагательным/Find antonyms for the adjectives:**
влажный (intense) – …, интенсивный (heavy) – …, постоянный (persistent) – …, небольшой (small) – …, редкий (rare) – …, слабый (slight) – …
**Exercise 2. Выразите несогласие/Express disagreement: ответьте отрицательно, используя антонимы пункта а)/Give negative answers, using the antonyms of a)**
1) При астме у больного сухой кашель? (Does a patient with asthma have a dry cough?) 2) При аллергии у пациента слабый кашель? (Does a patient with an allergy have a slight cough?) 3) При бронхите у больной кашель с мокротой? (Does the patient with bronchitis have a cough with sputum?) 4) При пневмонии у больного приступообразный кашель? (Does a patient with pneumonia have a paroxysmal cough?)
**Exercise 3. a) Найдите синонимы к прилагательным, используя слова задания 1/Find synonyms for the adjectives, using the words of Exercise 1**
интенсивный (heavy) – …, небольшой (slight) – …, влажный (wet) – …, приступами (paroxysmal) – …, постоянный – (persistent) …
**Exercise 4. Выразите согласие/Express agreement: ответьте положительно, используя синонимы упражнения 3/Give positive answers, using synonyms of exercise 3**
1) При астме у больного сильный кашель? (Does a patient with asthma have a bad cough?) 2) При простуде у пациента слабый кашель? (Does a patient with a cold have a slight cough?) 3) При пневмонии у больной кашель с мокротой? (Does a patient with pneumonia have a cough with sputum?) 4) При эмфиземе лёгких у пациента интенсивный кашель? (Does a patient with pulmonary emphysema have a heavy cough?)

**Table 5.** An example of exercises on collocations usage [28].

**Exercise 1. Составьте словосочетания/Make up word-combinations**		
**колоть что?/где? (to feel a shooting pain in where?)**		грудь (chest) – (в) левая часть (in the left side)
**давить что?/где? (to feel a tightness in where?)**		затылок (back of head), сердце (heart)
**ныть что?/где? (to ache; to feel an aching pain in where?)**		живот (belly) – (под) ложечка (in the pit of the stomach)
**резать что?/где? (to feel an acute pain in where?)**		живот (belly) – (в) правая часть (in the right side)
**сжимать (to feel a squeezing pain in …)**	**что? (-)**	голова (head), сердце (heart), затылок (back of head)
	**где? (where?)**	(в) грудная клетка (in the thoracic cage), (в) грудь (in the chest)

It is important to pay attention to the specific syntax of oral colloquial discourse and its differences from syntactic means used by students, for example, when they communicate with hospital physicians (Table 6).

**Table 6.** An example of exercises on speech transformations [28].

**Exercise 1. Трансформируйте запись истории болезни в реплики больного/Transform the records of the medical case history into the patient's remarks**
**Pattern:** Больной жалуется на общее недомогание (The patient complaints of general uneasiness). – Я чувствую себя плохо (I feel sick).
1) Больной жалуется на постоянный мучительный кашель (The patient complains of a persistent troublesome cough). 2) Больного беспокоит заложенность груди (The patient is worried about his/her chest congestion). 3) Больной жалуется на першение в горле, охриплость голоса, озноб (The patient complaints of the throat irriattion, hoarseness and chills). 4) Больного беспокоит потливость по ночам (The patient is worried about sweating at night).

After that, students are offered some typical samples of dialogue interviews, which reflect the studied discursive strategy. Then, international students acquire the skills to begin communication within a specified schedule, maintain and direct discursive interaction according to communicative goals etc (Table 7).

**Table 7.** An example of working out the interviewing tactics [28].

**Exercise 1. Составьте диалоги. Сообщите о физическом состоянии человека/Make up dialogues. Report the patient’s physical condition**			
1. – У вас озноб? (Do you feel feverish?) 2. – Да, меня знобит (Yes, I do.)		1) больной – лихорадка (patient – fever) 2) больная – тошнота (patient – nausea) 3) пациент – рвота (patient – vomiting)	
**Exercise 2. Составьте диалоги. Спросите о цвете, консистенции мокроты и о наличии крови/гноя в мокроте/Make up dialogues. Ask about the colour, consistence and the presence of blood/pus in sputum**			
**Disease**	**Sputum colour**	**Sputum consistency**	**Blood/pus in sputum**
**1) lobar pneumonia**	rusty	viscous	blood
**2) bronchial pneumonia**	yellow	mucous	pus
**3) pulmonary emphysema**	(from) grey (to) green	viscous and liquid	pus
**4) asthma**	transparent	globular	
5) pulmonary cancer	dark	mucous	pus, blood

### Involving a role-playing game

At the final stage, students are offered specific situations that recreate the conditions of real communication (the informative plan of discursive interaction can be presented in educational materials using fragments of real records in medical case histories, a thematic map of the situation, tables indicating the necessary information about the intended patient etc) (Table 8).

**Table 8.** Examples of role-playing situations [28].

**Situation 1.** Patient A. complains of asthma attacks. The attacks are accompanied by a dry cough, nasal stuffiness, chest congestion, and throat irritation. The asthma attacks have appeared recently (two months ago), after pneumonia. During an attack, sputum appears in small quantities. The patient coughs up mucus sputum of viscous consistency. The patient is worried about breathing difficulty between attacks. The patient has not taken medicinal drugs.
**Situation 2.** While visiting a gastroenterologist, patient D. complains of intense paroxysmal aching and drawing pains in the right hypochondrium, accompanied by flatulence. The pains occur after fried and spicy meals. The patient also complains of severe weakness, nausea on an empty stomach, persistent heartburn, and dry mouth. For heartburn, the patient takes "Renny" pills. The dietary pattern is neglected (junk food); poor appetite; irregular stools and diarrhea.

Thus, the skills and abilities required to implement the patient interviewing strategy and tactics are gradually developed at each stage of the proposed methodological algorithm. A similar strategy was presented by Heng Zhang *et al.*, where authors described the process of learning English through practicing speech in small groups [29].

## Material and Methods

This article describes the methodological strategy of teaching English to medical students. The main aim of this methodology is to improve professional (medical) conversational skills. Teaching foreign languages for medical purposes was tested during 2016–2018.

### Characteristics of participants

To confirm the effectiveness of the proposed methodological strategy for developing the skills and abilities in interviewing patients, the results of the testing, which was held at the Russian Language Department of the Medical Institute of the Peoples' Friendship University of Russia from 2016 to 2018, will be presented. Training in Group 1 was conducted during the academic year, four hours per week, corresponding to 136 classroom hours in groups of international students who studied Russian as a foreign language. To assess the effectiveness of the proposed model, we compared the results with Group 2, which attended a traditional course. This course did not include special tasks to form the skills in interviewing patients. Each group included 17 students in 2016, 16 students in 2017, and 15 students in 2018. The total number of students within these three years was 48 students in each group (first and second). The students were trained by professional teachers having at least ten years of experience in teaching Russian to foreigners. The starting level of Russian language proficiency was B1 in each group.

### Data sources and data collection methods

After the end of the training course, all the students participated in the speaking test that consisted of three tasks. To fulfill the first task, the students asked the patients questions, using the necessary tactics. The second task was to give answers to the doctor's statements. The last task was to dialogue with another student using a given situation. The materials were assessed following the scoring system recommended in the "The sample tests of Russian language as foreign (professional module)" [30]. The teachers evaluated the following items (Tables 9 and 10).

**Table 9.** Evaluation table for tasks 1 and 2.

**Position No.**	**Content (I)**			**Intention (II)**			**Total**
	**Adequately expressed**	**Expressed by inadequate means**	**Not expressed**	**Adequately expressed**	**Partially expressed**	**Not expressed**	**I + II**
**1**	2	1	0	2	1	0	
**2**	2	1	0	2	1	0	
**3**	2	1	0	2	1	0	
**4**	2	1	0	2	1	0	

**Table 10.** Evaluation table for Task 3.

**Monitor object**	**Evaluation items**	**Grading scale**						**Total**
**Content component**								
1. The ability to describe situations and actors:								
• completeness		0	1	2	3	4	5	
• accuracy		0	1	2	3	4	5	
**Intentions**								
2. The ability to express a request for information		0	1	2	3	4	5	
3. The ability to inform the interlocutor		0	1	2	3	4	5	
**Composition structure and form**								
4. The adequacy of the form and structure of the presentation to the content and intentions of the produced text				0	3	5		
**Linguistic means**								
5. Compliance with the norms of modern Russian language of the used:								
• lexical means		0	1	2				
• grammatical means		0	1	2				
• phonetic means		0	1	2				
• intonation means		0	1	2				

*The maximum score: 16 points for Tasks 1 and 2, and 33 points for Task 3. Total: 65 points.

### Data analysis procedures

The authors did the data analysis simultaneously with data collection. The teacher filled in the evaluation tables while talking to the students to collect qualitative and quantitative data. The spoken replies were recorded. Following this, the results were calculated, the percentage was derived, a qualitative analysis was conducted, and conclusions were made.

## Results

Table 11 presents the average results of both groups for 2016, 2017, and 2018. The average total score was calculated according to the formula: 
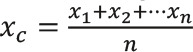
 [1].

**Table 11.** Average results for students' studying.

**2016**		**2017**		**2018**	
**Group 1**	**Group 2**	**Group 1**	**Group 2**	**Group 1**	**Group 2**
52/76.9%	34/52.3%	50/80%	33/50.8%	49/75.4%	36/55.4%

**Figure 1.**
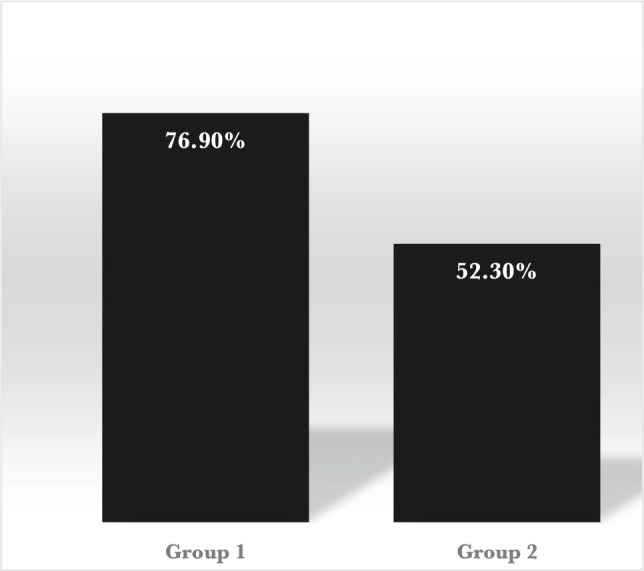
Dynamics of the results.

By calculating the average score for all 3 years for each group using a similar formula, it is possible to present the dynamics of the results for both groups (Figure 1).

As it can be seen from the diagram, the dynamics of the results show that the strategies used in Group 1 were more effective than the system applied in Group 2. 

In addition, a quantitative and qualitative analysis was conducted. While listening to students in Group 2, teachers noted they experienced some difficulties during the examination. When performing Task 1, the students sometimes had irrelevant answers, which confused them. As for Task 2, many tactics were expressed by inadequate means, which could distort the meanings of statements. In Task 3, while questioning the patient, the students were lost, forgot to clarify the necessary information, and asked questions without syntactic transformations, simply copying the information from the dialogue descriptions presented to them.

Additionally, they forgot important questions that affect the diagnosis. These inaccuracies impact the doctor's ability to fully interrogate the patient; it might alter the meaning of comments or cause a complete misunderstanding of the received information. Comparing students' answers from Group 1, the teachers noted that they usually did not make errors of this kind. Nevertheless, some communicatively insignificant errors that did not affect the meanings of statements were observed.

## Discussion

The current study demonstrates the efficacy of the proposed methodological model. We would also like to point out that a similar technique may be applied to educate students on interviewing a patient with stomatological diseases. Of course, the method needs to be modified because the specifics of interviewing such a patient and the language means by which the interviewing strategy is implemented would be different. It is also worth noting that another technique for teaching future doctors to communicate with colleagues could be developed. In future scientific studies, some new points may be added and discussed. One more thing to be included in future training is analyzing common mistakes made by the doctors while interviewing a patient. It may decrease the patient's trust and lead to some medical errors. It is possible to arrange some special cases to be included to avoid such kinds of mistakes.

## Conclusion

Interviewing patients is one of the most important activities in terms of a correct diagnosis and, as a result, patient recovery. When teaching a professional medical language to international students, teachers should pay special attention to this strategy and its tactics.

The presented algorithm for teaching patient interviewing strategy consists of three parts: (1) introducing a typical staged interviewing strategy; (2) acquiring necessary linguistic means of implementing the interviewing strategy; and (3) involving a role-playing game to imitate real communication between the doctor and the patient. Each part contains a certain methodological content: types of tasks that can form the skills in interviewing patients.

During 2016–2018, this methodology was tested. The positive results allow the authors to speak about the effectiveness of this training and consider it a possible option for teaching international medical students the essential component of their professional and communicative competence, *i.e.,* patient interviewing strategies.

## Acknowledgments

### Conflict of interest

The authors declare no conflict of interest.

### Ethical approval

The study was approved by the Ethical Committee of the Medical Institute of RUDN University (approval number No.4, December 20, 2018).

### Consent to participate

Written informed consent was obtained from the participants.

### Data availability

Further data is available from the corresponding author on reasonable request.

### Funding

The publication has been prepared with the support of the RUDN University Program 5-100.

### Authorship

VK contributed to conceptualizing, methodology, and critical review. YB contributed to writing the original draft, analysis, and interpretation of the data. YG contributed to data collection and data processing. KK contributed to the literature review analysis. MB contributed to editing the manuscript, analysis, and interpretation of the data.
